# Supplementary material for: Characteristics and Discrimination of the Commercial Chinese Four Famous Vinegars Based on Flavor Compositions
Source: Foods. 2023 Apr 30;12(9):1865. doi: 10.3390/foods12091865 (PMC10178022; doi:10.3390/foods12091865)
Supplement: Supplementary file 1 [file foods-12-01865-s001.zip › foods-2289800-supplementary.pdf]

Total content (mg/mL)				
	SAV (n=8)	ZAV (n=8)	FMV (n=8)	SBV (n=8)
Polyphenol	245668.75 ± 11550.12^{b}	281811.3 ± 9063.78^{b}	7388.8 ± 1518.68^{a}	39262.5 ± 9520.75^{c}
Flavone	254.03 ± 117.79^{b}	224.02 ± 93.70^{b}	65.13 ± 9.37^{a}	310.62 ± 98.81^{b}

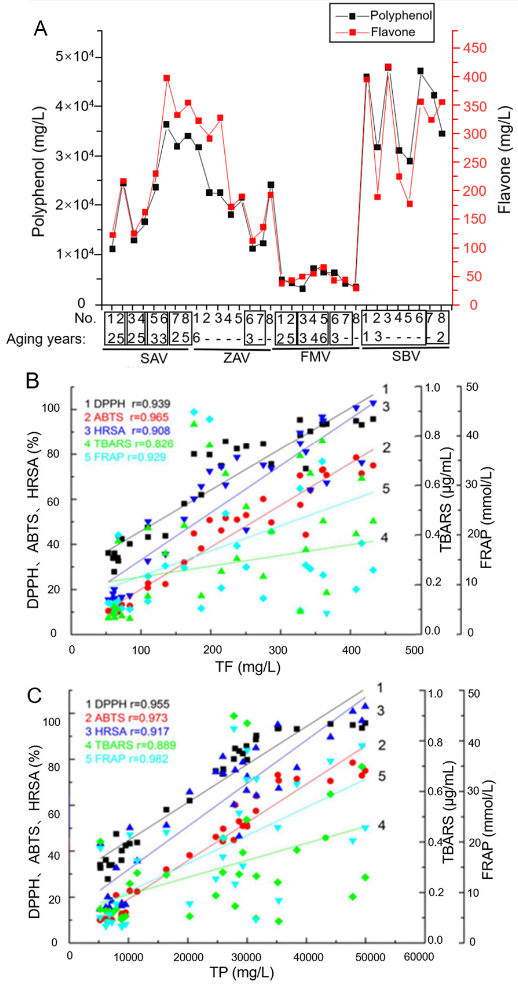

Figure S1 The antioxidant capacity analysis for SAV, ZAV, FMV and SBV. (A) The contents of TF and TP in samples. The sample numbers and aging years were marked at the bottom. The samples in the box were from the same brand. Contents of TF and TP were depicted on top. Different letters indicated significant difference, $p < 0.05$. (B) The correlation between 5 TACs (ABTS, DPPH, FRAP, TBARS and HRSA) and

TF. (C) The correlation between 5 TACs (ABTS, DPPH, FRAP, TBARS and HRSA) and TP.

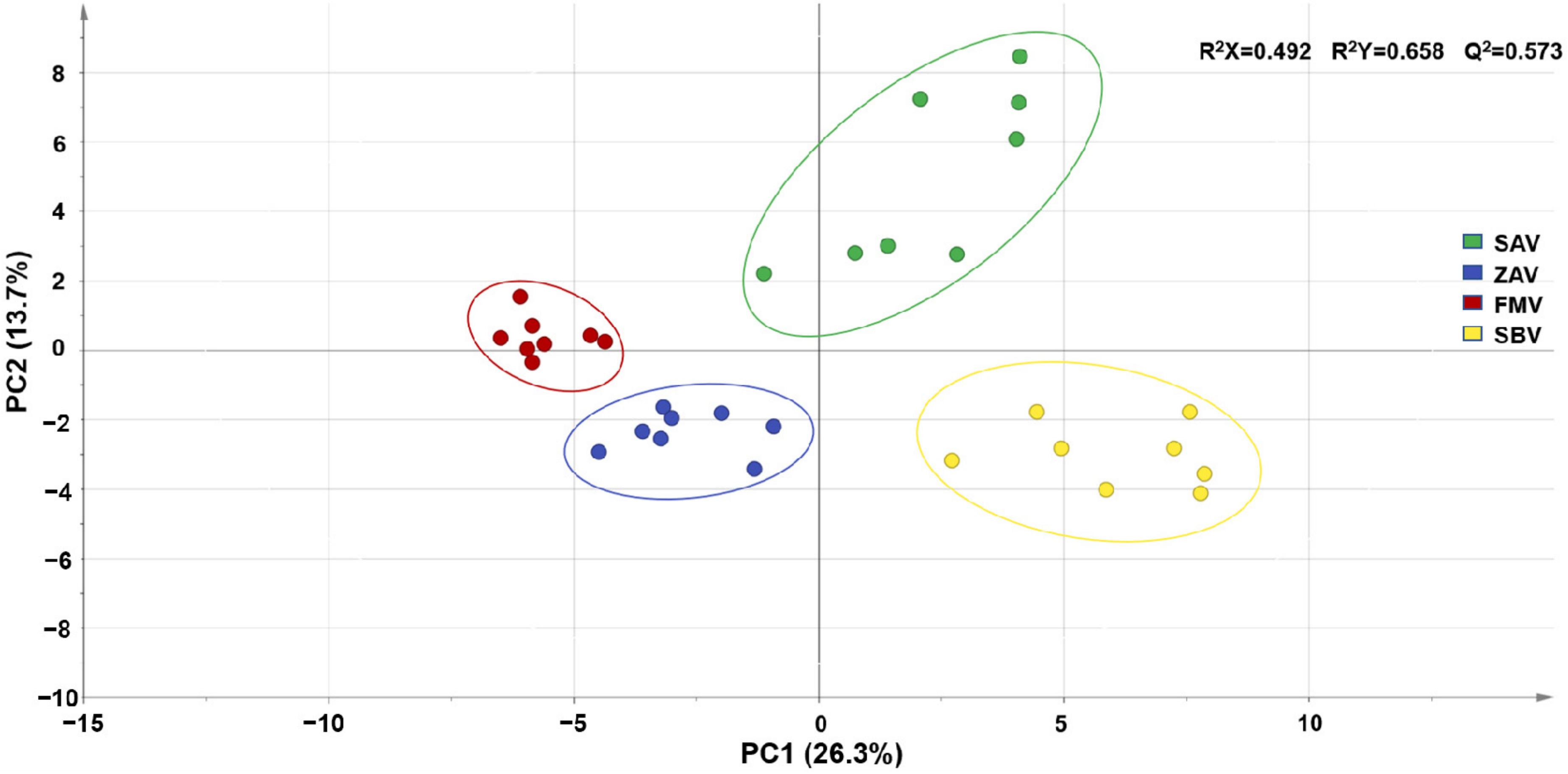

Figure S2 The OPLS-DA analysis for SAV, ZAV, FMV, and SBV based on 68 volatiles, amino acids and organic acids.

Table S1 The informations of the samples of SAV, ZAV, FMV and SBV.

Samples	Manufacturer	Producing area	Aging years
SAV1	Shanxi Water Tower Vinegar Co., Ltd.	Taiyuan City, Shanxi Province	2
SAV2	Shanxi Water Tower Vinegar Co., Ltd.	Taiyuan City, Shanxi Province	5
SAV3	Shanxi Zilin Vinegar Industry Co., Ltd.	Taiyuan City, Shanxi Province	2
SAV4	Shanxi Zilin Vinegar Industry Co., Ltd.	Taiyuan City, Shanxi Province	5
SAV5	Shanxi Fuyuan Chang Old vinegar Co., Ltd.	Taiyuan City, Shanxi Province	3
SAV6	Shanxi Fuyuan Chang Old vinegar Co., Ltd.	Taiyuan City, Shanxi Province	3
SAV7	Taiyuan Ninghuafu Yiyuanqing Vinegar Co., Ltd.	Taiyuan City, Shanxi Province	2
SAV8	Taiyuan Ninghuafu Yiyuanqing Vinegar Co., Ltd.	Taiyuan City, Shanxi Province	5
ZAV1	Jiangsu Hengshun Vinegar Co., Ltd.	Zhenjiang City, Jiangsu Province	6
ZAV2	Jiangsu Hengshun Vinegar Co., Ltd.	Zhenjiang City, Jiangsu Province	-
ZAV3	Jiangsu Hengshun Vinegar Co., Ltd.	Zhenjiang City, Jiangsu Province	-
ZAV4	Jiangsu Hengshun Vinegar Co., Ltd.	Zhenjiang City, Jiangsu Province	-
ZAV5	Jiangsu Hengshun Vinegar Co., Ltd.	Zhenjiang City, Jiangsu Province	-
ZAV6	Jiangsu Hengkang Seasoning Factory	Zhenjiang City, Jiangsu Province	3
ZAV7	Jiangsu Hengkang Seasoning Factory	Zhenjiang City, Jiangsu Province	-
ZAV8	Zhenjiang Hengfeng Sauce and Vinegar Co., Ltd.	Zhenjiang City, Jiangsu Province	-
FMV1	Fujian Yongchun Vinegar Vinegar Industry Co., Ltd.	Quanzhou City, Fujian Province	2
FMV2	Fujian Yongchun Vinegar Vinegar Industry Co., Ltd.	Quanzhou City, Fujian Province	5
FMV3	Fujian Yongchun Jinchun Brewing Co., Ltd.	Quanzhou City, Fujian Province	3
FMV4	Fujian Yongchun Jinchun Brewing Co., Ltd.	Quanzhou City, Fujian Province	4
FMV5	Fujian Yongchun Jinchun Brewing Co., Ltd.	Quanzhou City, Fujian Province	6
FMV6	Yongchun County Yongchun Vinegar Co., Ltd.	Quanzhou City, Fujian Province	3
FMV7	Yongchun County Yongchun Vinegar Co., Ltd.	Quanzhou City, Fujian Province	-
FMV8	Fujian Quanzhou Fuquanchun Food Co., Ltd.	Quanzhou City, Fujian Province	-
SBV1	Sichuan Baoning Vinegar Co., Ltd	Nanchong City, Sichuan Province	1
SBV2	Sichuan Baoning Vinegar Co., Ltd	Nanchong City, Sichuan Province	3
SBV3	Sichuan Baoning Vinegar Co., Ltd	Nanchong City, Sichuan Province	-
SBV4	Sichuan Baoning Vinegar Co., Ltd	Nanchong City, Sichuan Province	-

SBV5	Sichuan Baoning Vinegar Co., Ltd	Nanchong City, Sichuan Province	-
SBV6	Sichuan Baoning Vinegar Co., Ltd	Nanchong City, Sichuan Province	-
SBV7	Sichuan Yuzhong Vinegar Industry Co., Ltd.	Nanchong City, Sichuan Province	-
SBV8	Sichuan Yuzhong Vinegar Industry Co., Ltd.	Nanchong City, Sichuan Province	2

-, aging year was not indicated on the product label.

Table S2 The volatiles in SAV, ZAV, FMV and SBV.

Compounds	RI	Characteristic ion[B] (m/z)	SAV (n=8)		ZAV (n=8)		FMV (n=8)		SBV (n=8)	
			Share[C]	Contents [A]	Share	Contents [A]	Share	Contents [A]	Share	Contents [A]
Esters										
**3-Methylbutyl acetate**	**540**	**43 (70, 55)**	**3/8**	**355.40 ± 520.20[b]**	**5/8**	**1165.30 ± 1247.70[b]**	**8/8**	**8860.10 ± 3312.20[a]**	**0/8**	**0.00 ± 0.00[b]**
**Ethyl Acetate**	**803**	**61 (43, 45)**	**8/8**	**1250.20 ± 1365.60[b]**	**8/8**	**1161.80 ± 1397.00[b]**	**8/8**	**3914.90 ± 2217.90[a]**	**8/8**	**593.70 ± 364.91[b]**
Hexanoic acid ethyl ester	842	88 (99, 43)	2/8	43.60 ± 80.90[a]	0/8	0.00 ± 0.00[a]	6/8	39.95 ± 29.46[a]	0/8	0.00 ± 0.00[a]
Acetic acid hexyl ester	897	43 (56, 69, 84)	1/8	20.55 ± 58.12[ab]	5/8	74.75 ± 93.65[a]	1/8	1.71 ± 4.84[b]	0/8	0.00 ± 0.00[b]
Ethyl L(-)-lactate	953	45 (75)	0/8	0.00 ± 0.00[b]	4/8	74.80 ± 80.72[b]	1/8	35.55 ± 100.55[b]	5/8	211.15 ± 189.26[a]
2-Furanmethanol acetate	1073	81 (43, 98, 140)	5/8	189.15 ± 310.24[a]	6/8	74.90 ± 52.07[ab]	0/8	0.00 ± 0.00[b]	2/8	27.55 ± 57.24[ab]
Ethyl 2-hydroxyisocaproate	1078	69 (87,43)	0/8	0.00 ± 0.00[b]	3/8	71.35 ± 99.56[a]	1/8	8.00 ± 22.62[b]	0/8	0.00 ± 0.00[b]
**Diethyl butanodiate**	**1141**	**101 (129, 74, 55)**	**6/8**	**74.60 ± 53.90[a]**	**8/8**	**678.70 ± 1019.10[a]**	**5/8**	**475.60 ± 1057.60[a]**	**1/8**	**13.90 ± 39.20[a]**
Acetic acid benzyl ester	1164	108 (91, 43)	1/8	9.15 ± 25.88[a]	0/8	0.00 ± 0.00[a]	0/8	0.00 ± 0.00[a]	0/8	0.00 ± 0.00[a]
**2-Methylpropyl acetate**	**1173**	**43 (56, 73)**	**0/8**	**0.00 ± 0.00[b]**	**0/8**	**0.00 ± 0.00[b]**	**8/8**	**892.70 ± 289.70[a]**	**0/8**	**0.00 ± 0.00[b]**
**Ethyl phenylacetate**	**1188**	**91 (164, 65)**	**6/8**	**46.70 ± 32.60[b]**	**8/8**	**83.70 ± 30.50[a]**	**8/8**	**53.20 ± 39.30[ab]**	**5/8**	**44.20 ± 39.20[b]**
2-Phenylethyl propionate	1202	104 (43, 91)	1/8	120.80 ± 341.67[a]	2/8	407.55 ± 755.12[a]	1/8	361.35 ± 1022.05[a]	1/8	85.45 ± 241.68[a]
Acetic acid 2-phenylethyl ester	1208	104 (43, 91)	3/8	290.30 ± 401.90[a]	6/8	1701.00 ± 1224.10[a]	4/8	1954.87 ± 3131.62[a]	6/8	497.00 ± 364.67[a]
Ethyl hexadecanoate	1456	88 (44, 73, 101)	1/8	0.60 ± 1.69[a]	3/8	3.35 ± 4.87[a]	1/8	0.16 ± 0.47[a]	3/8	2.30 ± 3.90[a]
Pyrazines										
Methylpyrazine	901	94 (67, 40)	1/8	8.35 ± 23.61[a]	0/8	0.00 ± 0.00[a]	2/8	14.55 ± 38.78[a]	0/8	0.00 ± 0.00[a]
2,6-Dimethylpyrazine	946	108 (42, 39)	0/8	0.00 ± 0.00[a]	0/8	0.00 ± 0.00[a]	1/8	4.75 ± 13.43[a]	0/8	0.00 ± 0.00[a]
2,3-Dimethylpyrazine	958	108 (67, 40)	3/8	41.50 ± 80.58[a]	0/8	0 ± 0[a]	1/8	1.30 ± 3.67[a]	1/8	4.80 ± 13.57[a]
**2,3,5-Trimethylpyrazine**	**1002**	**122 (42, 81)**	**8/8**	**261.05 ± 140.70[a]**	**2/8**	**3.20 ± 6.10[b]**	**3/8**	**22.60 ± 39.00[b]**	**8/8**	**63.80 ± 74.70[b]**
2,3-Dimethyl-5-ethylpyrazine	1033	135 (96, 39)	1/8	295.50 ± 835.80[a]	0/8	0.00 ± 0.00[a]	0/8	0.00 ± 0.00[a]	0/8	0.00 ± 0.00[a]
**2,3,5,6-Tetramethylpyrazine**	**1041**	**136 (54, 42)**	**8/8**	**886.20 ± 936.10[a]**	**0/8**	**0.00 ± 0.00[b]**	**1/8**	**2.00 ± 5.70[b]**	**8/8**	**196.21 ± 247.30[b]**
2,3,5-Trimethyl-6-ethylpyrazine	1064	149 (81, 43)	2/8	34.10 ± 63.20[a]	0/8	0.00 ± 0.00[a]	0/8	0.00 ± 0.00[a]	0/8	0.00 ± 0.00[a]
Alcohols										
Eucalyptol	811	111 (43, 55)	2/8	143.15 ± 265.94[a]	0/8	0.00 ± 0.00[a]	0/8	0.00 ± 0.00[a]	0/8	0.00 ± 0.00[a]

Benzyl alcohol	1066	106 (77, 51)	2/8	29.20 ± 70.00^{a}	0/8	0.00 ± 0.00^{a}	0/8	0.00 ± 0.00^{a}	0/8	0.00 ± 0.00^{a}
**2,3-Butanediol**	**1098**	**45 (57)**	**3/8**	**142.70 ± 230.10^{ab}**	**8/8**	**288.10 ± 151.00^{a}**	**3/8**	**44.30 ± 63.30^{b}**	**2/8**	**84.20 ± 160.60^{b}**
**Phenylethyl alcohol**	**1127**	**91 (120, 65)**	**8/8**	**675.50 ± 265.10^{b}**	**8/8**	**2082.00 ± 409.20^{a}**	**8/8**	**2579.20 ± 1524.60^{a}**	**5/8**	**635.20 ± 636.90^{b}**
2-Furanmethanol	1134	98 (81, 91)	4/8	345.15 ± 713.85^{a}	0/8	0.00 ± 0.00^{a}	0/8	0.00 ± 0.00^{a}	0/8	0.00 ± 0.00^{a}
Acids										
2-Ketobutan-3-yl acetate	978	43 (87)	3/8	52.00 ± 87.13^{a}	4/8	39.30 ± 42.43^{a}	2/8	35.75 ± 67.21^{a}	0/8	0.00 ± 0.00^{a}
**Acetic acid**	**1016**	**43 (60)**	**8/8**	**16577.25 ± 5738.30^{a}**	**8/8**	**12546.90 ± 1770.50^{b}**	**8/8**	**16724.40 ± 7989.00^{a}**	**8/8**	**8362.90 ± 1919.00^{c}**
Propanoic acid	1074	74 (45, 57)	4/8	69.50 ± 76.82^{a}	0/8	0.00 ± 0.00^{b}	3/8	39.85 ± 59.74^{ab}	2/8	30.75 ± 69.26^{ab}
2-Methylpropanoic acid	1092	43 (73, 88)	2/8	27.90 ± 52.00^{a}	2/8	28.10 ± 52.42^{a}	3/8	48.40 ± 68.37^{a}	2/8	32.05 ± 66.51^{a}
Butanoic acid	1121	60 (73, 43)	1/8	17.35 ± 49.07^{b}	0/8	0.00 ± 0.00^{b}	0/8	0.00 ± 0.00^{b}	4/8	100.70 ± 127.03^{a}
**3-Methylbutanoic acid**	**1138**	**60 (43, 87)**	**5/8**	**401.60 ± 442.00^{a}**	**8/8**	**511.50 ± 235.00^{a}**	**6/8**	**254.90 ± 239.10^{a}**	**8/8**	**501.60 ± 357.30^{a}**
Pentanoic acid	1168	60 (73, 41)	2/8	55.55 ± 118.42^{a}	1/8	3.45 ± 9.75^{a}	1/8	15.35 ± 43.41^{a}	3/8	22.80 ± 34.31^{a}
**Hexanoic acid**	**1219**	**60 (73, 41)**	**8/8**	**493.25 ± 430.00^{a}**	**8/8**	**205.10 ± 53.00^{b}**	**8/8**	**100.90 ± 25.30^{c}**	**8/8**	**526.20 ± 473.40^{a}**
**Octanoic acid**	**1347**	**60 (73, 43)**	**6/8**	**42.35 ± 30.60^{b}**	**5/8**	**21.30 ± 19.50^{b}**	**8/8**	**92.60 ± 50.40^{a}**	**2/8**	**16.70 ± 31.40^{b}**
4-Hexyl-2,5-dioxofuran-3-acetic acid	1370	126 (40, 98)	0/8	0.00 ± 0.00^{b}	0/8	0.00 ± 0.00^{b}	4/8	58.80 ± 102.59^{a}	0/8	0.00 ± 0.00^{b}
Furans										
**Furfural**	**1032**	**96 (39)**	**8/8**	**14145.10 ± 7558.00^{b}**	**8/8**	**22370.60 ± 10740.60^{a}**	**8/8**	**564.40 ± 331.40^{c}**	**8/8**	**21560.50 ± 4936.90^{a}**
1-(2-furanyl)-Ethanone	1055	95 (110)	7/8	153.95 ± 79.22^{a}	3/8	30.90 ± 42.90^{b}	2/8	15.20 ± 28.19^{b}	4/8	44.15 ± 47.5^{b}
2,2-Methylenebisfuran	1112	91 (148, 120)	1/8	10.55 ± 29.83^{a}	6/8	44.80 ± 111.26^{a}	0/8	0.00 ± 0.00^{a}	2/8	5.20 ± 10.08^{a}
2-Acetyl-5-methylfuran	1115	91 (108, 43, 123)	2/8	8.30 ± 18.10^{a}	0/8	0.00 ± 0.00^{a}	0/8	0.00 ± 0.00^{a}	0/8	0.00 ± 0.00^{a}
Phenols										
2-Methoxyphenol	1226	109 (124, 81)	6/8	115.80 ± 98.81^{a}	1/8	2.65 ± 7.49^{b}	0/8	0.00 ± 0.00^{b}	2/8	8.60 ± 17.97^{b}
**2-methoxy-4-methylphenol**	**1280**	**138 (123, 95)**	**7/8**	**345.50 ± 336.60^{a}**	**8/8**	**88.30 ± 26.80^{bc}**	**4/8**	**9.80 ± 13.70^{c}**	**8/8**	**221.30 ± 76.20^{ab}**
2-Methoxy-5-methylphenol	1281	138 (123, 95, 60)	0/8	0.00 ± 0.00^{a}	1/8	8.65 ± 24.46^{a}	2/8	9.05 ± 17.37^{a}	0/8	0.00 ± 0.00^{a}
2-Methylphenol	1307	123 (108, 44)	2/8	11.60 ± 21.63^{a}	1/8	0.60 ± 1.69^{a}	0/8	0.00 ± 0.00^{a}	5/8	5.65 ± 6.69^{a}
Phenol	1309	94 (66, 40)	3/8	13.55 ± 23.92^{a}	0/8	0.00 ± 0.00^{b}	1/8	0.75 ± 2.12^{b}	0/8	0.00 ± 0.00^{b}
4-Ethyl-2-methoxyphenol	1327	137 (152, 85)	4/8	152.65 ± 210.14^{a}	0/8	0.00 ± 0.00^{b}	5/8	72.10 ± 65.83^{ab}	1/8	35.55 ± 100.55^{ab}
4-Ethylphenol	1410	107 (122, 44)	6/8	31.30 ± 32.33^{a}	2/8	2.50 ± 4.64^{b}	3/8	12.05 ± 17.23^{b}	0/8	0.00 ± 0.00^{b}
2-Ethylphenol	1410	107 (122, 44)	1/8	2.35 ± 6.64^{a}	0/8	0.00 ± 0.00^{a}	1/8	1.70 ± 4.80^{a}	0/8	0.00 ± 0.00^{a}

Aldehydes									
**Nonanal**	**986**	**57 (43, 70, 98)**	**8/8**	**91.60 ± 38.70^{a}**	**5/8**	**38.80 ± 42.10^{b}**	**4/8**	**40.20 ± 45.40^{b}**	**6/8**	**36.10 ± 28.90^{b}**
**Benzaldehyde**	**1066**	**106 (77, 51)**	**8/8**	**1249.25 ± 457.50^{a}**	**8/8**	**567.40 ± 296.40^{b}**	**8/8**	**449.50 ± 449.50^{b}**	**8/8**	**514.40 ± 150.20^{b}**
**5-Methyl-2-furancarboxaldehyde**	**1095**	**110 (53, 43, 81)**	**8/8**	**767.55 ± 404.30^{a}**	**2/8**	**20.80 ± 42.30^{c}**	**3/8**	**88.80 ± 168.80^{c}**	**8/8**	**335.90 ± 151.80^{b}**
Beta-cyclocitral	1119	137 (152, 109, 40)	0/8	0.00 ± 0.00^{b}	4/8	12.10 ± 13.360^{a}	0/8	0.00 ± 0.00^{b}	0/8	0.00 ± 0.00^{b}
Benzeneacetaldehyde	1127	91 (120, 65)	3/8	27.50 ± 46.37^{b}	5/8	94.85 ± 84.89^{a}	7/8	31.50 ± 32.24^{b}	2/8	25.40 ± 47.65^{b}
2-Methyl-3(2-furyl)acrolein	1235	79 (136, 40, 108)	5/8	30.65 ± 31.38^{ab}	2/8	5.00 ± 9.87^{bc}	0/8	0.00 ± 0.00^{c}	7/8	48.40 ± 43.64^{a}
**2-Phenylcrotonaldehyde**	**1267**	**115 (146, 91)**	**8/8**	**82.15 ± 43.1^{a}**	**8/8**	**44.60 ± 19.60^{bc}**	**6/8**	**32.90 ± 36.00^{c}**	**8/8**	**69.50 ± 26.30^{ab}**
1H-Pyrrole-2-carboxaldehyde	1325	95 (66, 39, 135)	4/8	28.65 ± 31.23^{a}	2/8	4.35 ± 8.30^{b}	0/8	0.00 ± 0.00^{b}	0/8	0.00 ± 0.00^{b}
**5-Methyl-2-phenyl-2-hexenal**	**1355**	**107 (103, 90, 188)**	**8/8**	**36.40 ± 24.90^{b}**	**7/8**	**26.50 ± 16.20^{b}**	**0/8**	**0.00 ± 0.00^{b}**	**8/8**	**97.10 ± 61.90^{a}**
Butanal		43 (72, 206)	0/8	0.00 ± 0.00^{a}	0/8	0.00 ± 0.00^{a}	0/8	0.00 ± 0.00^{a}	2/8	1.97 ± 4.26^{a}
Ketones										
3-Hydroxy-2-butanone	914	45 (43, 88)	5/8	310.70 ± 362.05^{a}	7/8	555.35 ± 289.21^{a}	7/8	633.65 ± 360.68^{a}	3/8	258.75 ± 370.39^{a}
1,3,3-Trimethyl-bicyclo-heptan-2-one	992	81 (69, 41)	3/8	43.25 ± 86.19^{a}	0/8	0.00 ± 0.00^{a}	0/8	0.00 ± 0.00^{a}	0/8	0.00 ± 0.00^{a}
4-(2-furanyl)-3-Buten-2-one	1253	121 (40, 136, 65)	1/8	0.60 ± 1.69^{b}	6/8	6.05 ± 6.52^{a}	0/8	0.00 ± 0.00^{b}	0/8	0.00 ± 0.00^{b}
**Dihydro-5-pentyl-2(3H)-furanone**	**1329**	**85 (56, 41)**	**0/8**	**0.00 ± 0.00^{b}**	**8/8**	**132.00 ± 39.80^{a}**	**1/8**	**4.70 ± 13.30^{b}**	**4/8**	**110.70 ± 127.20^{a}**
Others										
Styrene	878	104 (78, 40)	3/8	13.20 ± 18.23^{a}	1/8	1.80 ± 3.65^{a}	6/8	15.05 ± 12.69^{a}	2/8	5.82 ± 14.20^{a}
1,2,3,4-Tetramethylbenzene	1047	119 (134, 91,39)	0/8	0.00 ± 0.00^{a}	1/8	9.30 ± 26.30^{a}	0/8	0.00 ± 0.00^{a}	1/8	25.50 ± 72.12^{a}
1,2,3,5-Tetramethylbenzene	1048	119 (134, 95,39)	0/8	0.00 ± 0.00^{b}	5/8	111.35 ± 132.32^{a}	0/8	0.00 ± 0.00^{b}	4/8	63.95 ± 94.06^{ab}
4-Ethyl-1,3-benzenediol	1167	123 (138, 43)	3/8	19.10 ± 33.22^{a}	0/8	0.00 ± 0.00^{b}	0/8	0.00 ± 0.00^{b}	0/8	0.00 ± 0.00^{b}
**1,2-Dihydro-1,1,6-trimethylnaphthalene**	**1172**	**157 (142, 172)**	**1/8**	**10.30 ± 29.13^{b}**	**8/8**	**61.05 ± 33.44^{b}**	**1/8**	**2.10 ± 5.93^{b}**	**8/8**	**176.45 ± 102.69^{a}**
1-(1H-pyrrol-2-yl)-Ethanone	1290	94 (109, 40)	6/8	14.75 ± 11.62^{a}	2/8	1.90 ± 3.91^{b}	0/8	0.00 ± 0.00^{b}	5/8	8.27 ± 10.14^{ab}
Total				40788.98 ± 6861.95^{a}		45281.9 ± 8145.61^{a}		37968.20 ± 7413.49^{a}		35415.16 ± 7618.91^{a}

The compounds were grouped according to the chemical structure as esters (14), pyrazines (7), alcohols (5), acids (10), furans (4), phenols (8), aldehydes (10), ketones (4) and others (6). [A]The relative peak area of compounds against internal parameters (2-octanol). [B]The ion used for compound identification. [C]The numbers of samples in which the substance was detected. Each type of vinegars contain 8 samples. Different superscript lowercase letters indicate ignificant difference in the

same row, $p < 0.05$. Bold, the compounds are shared by all samples of at least one type of vinegar.

Table S3 The distribution of volatile variety numbers in volatile groups of SAV, ZAV, FMV and SBV.

Compound classes	Variety			
	SAV (n=8)	ZAV (n=8)	FMV (n=8)	SBV (n=8)
Esters	4.62 ± 1.59^{b}	7.25 ± 0.89^{a}	6.50 ± 1.19^{a}	3.50 ± 1.60^{b}
Pyrazines	2.87 ± 0.99^{a}	0.125 ± 0.35^{b}	1.00 ± 1.51^{b}	2.12 ± 0.35^{a}
Alcohols	2.37 ± 1.06^{a}	2.0 ± 0^{ab}	1.37 ± 0.52^{bc}	0.87 ± 0.64^{c}
Acids	4.87 ± 1.30^{a}	4.5 ± 1.07^{a}	5.37 ± 1.18^{a}	4.60 ± 1.59^{a}
Furans	2.25 ± 0.71^{a}	2.12 ± 0.83^{a}	1.25 ± 0.46^{b}	1.75 ± 0.71^{ab}
Phenols	3.62 ± 1.30^{a}	1.62 ± 0.74^{b}	2.00 ± 0.92^{b}	2.00 ± 0.53^{b}
Aldehydes	6.5 ± 0.53^{a}	5.37 ± 1.40^{a}	3.5 ± 0.92^{b}	6.12 ± 1.13^{a}
Ketones	1.12 ± 0.35^{b}	2.60 ± 0.74^{a}	1.00 ± 0.53^{b}	0.87 ± 0.64^{b}
Others	1.63 ± 1.06^{ab}	1.75 ± 1.06^{ab}	0.88 ± 1.01^{b}	2.55 ± 1.24^{a}
Total	29 ± 2.09^{a}	25 ± 2.23^{a}	22 ± 2.31^{a}	23 ± 1.87^{a}

Different superscript letters indicate significant difference in the same row, $p < 0.05$.

Table S4 The distribution of volatile contents in volatile groups of SAV, ZAV, FMV and SBV.

Compound classes	Relative percentage (%) (Range (%))[1]			
	SAV (n=8)	ZAV (n=8)	FMV (n=8)	SBV (n=8)
Esters	5.87 ± 3.81^{b} (2.16-12.85)	12.06 ± 6.09^{b} (6.48-21.78)	42.03 ± 19.04^{a} (8.75-65.61)	4.13 ± 3.00^{b} (1.15-10.37)
Pyrazines	3.73 ± 3.51^{a} (0.50-8.84)	0.01 ± 0.01^{b} (0.00-0.03)	0.12 ± 0.18^{b} (0.00-0.47)	0.74 ± 0.93^{b} (0.32-3.05)
Alcohols	3.27 ± 2.40^{bc} (0.70-8.27)	5.20 ± 0.97^{ab} (3.66-6.51)	6.90 ± 3.89^{a} (2.53-12.14)	2.01 ± 1.76^{c} (0.00-2.57)
Acids	43.48 ± 7.01^{a} (34.37-55.11)	29.49 ± 3.85^{b} (22.89-34.73)	45.69 ± 8.56^{a} (32.42-56.16)	26.85 ± 7.82^{b} (19.73-44.43)
Furans	35.02 ± 18.53^{b} (11.27-58.99)	49.25 ± 13.84^{a} (27.10-64.21)	1.52 ± 0.91^{c} (0.78-3.01)	60.48 ± 13.79^{a} (40.89-78.08)
Phenols	1.65 ± 1.59^{a} (0.04-4.05)	0.23 ± 0.07^{b} (0.14-0.34)	0.28 ± 0.21^{b} (0.02-0.57)	0.76 ± 0.45^{b} (0.31-1.73)
Aldehydes	5.66 ± 1.68^{a} (3.08-7.54)	1.79 ± 0.60^{c} (1.24-2.89)	1.69 ± 0.45^{c} (1.17-2.39)	3.15 ± 0.56^{b} (2.16-3.76)
Ketones	0.87 ± 0.84^{a} (0.01-2.10)	1.52 ± 0.67^{a} (0.24-2.50)	1.68 ± 0.97^{a} (0.00-2.86)	1.03 ± 1.01^{a} (0.00-2.52)
Others	0.24 ± 0.41^{b} (0.00-1.26)	0.41 ± 0.33^{ab} (0.03-0.99)	0.05 ± 0.10^{b} (0.00-0.32)	0.79 ± 0.63^{a} (0.16-2.00)

[1]The proportion of volatile contents in each group to the total contents of a certain kind of vinegar. Different superscript letters indicate significant difference in the same row, $p < 0.05$.

Table S5 The relative percentage of phenolic contents in SAV, ZAV, FMV and SBV.

Compounds	Relative percentage (%) (Range(%))			
	SAV (n=8)	ZAV (n=8)	FMV (n=8)	SBV (n=8)
Catechin	26.91 ± 6.50^{a} (0-27.17)	47.16 ± 9.00^{a} (46.05-48.32)	98.16 ± 9.70^{a} (93.53-99.3)	72.72 ± 6.64^{a} (72.10-73.33)
Gallic acid	21.20 ± 13.60^{b} (0-21.62)	5.36 ± 1.45^{b} (0-5.70)	-	27.28 ± 4.37^{b} (0-27.62)
p-coumaric acid	34.00 ± 7.81^{c} (0-34.05)	-	1.84 ± 1.02^{b} (0-1.96)	-
Ferulic acid	4.69 ± 2.10^{d} (0-4.84)	14.93 ± 7.90^{c} (0-16.34)	-	-
Caffeic acid	2.01 ± 1.01^{e} (0-2.08)	7.07 ± 6.81^{d} (0-7.36)	-	-
Chlorogenic acid	11.20 ± 7.10^{f} (0-11.44)	25.48 ± 14.60^{e} (0-26.03)	-	-
Rutin	12.01 ± 7.96^{g} (0-12.23)	13.88 ± 7.01^{f} (0-12.63)	-	-

-, not detected in vinegars. Different superscript letters indicate significant difference in the same row, $p < 0.05$.

Table S6 The relative percentage of free amino acid contents in SAV, ZAV, FMV and SBV.

Free amino acids	Relative percentage (%) (Range (%))			
	SAV (n=8)	ZAV (n=8)	FMV (n=8)	SBV (n=8)
Asp	5.94 ± 0.59^{b} (5.16-6.91)	5.21 ± 1.27^{b} (3.67-7.59)	3.24 ± 0.32^{a} (2.820-3.660)	4.0 ± 1.10^{a}(2.96-6.50)
Glu	8.79 ± 0.94^{bc} (7.69-10.32)	6.00 ± 1.02^{a} (4.31-7.81)	10.43 ± 4.17^{c} (5.61-17.42)	7.72 ± 1.65^{ab} (5.08-10.23)
Thr	4.23 ± 0.30^{a} (3.78-4.55)	4.09 ± 0.40^{a} (3.51-4.65)	4.18 ± 0.64^{a} (3.31-5.46)	4.24 ± 0.27^{a} (3.93-4.68)
Ala	8.51 ± 1.47^{a}(5.01-9.57)	11.84 ± 1.63^{b} (9.48-15.11)	11.24 ± 2.12^{b} (8.23-13.94)	12.82 ± 2.84^{b} (9.16-16.80)
Gly	4.64 ± 4.23^{a} (2.71-14.93)	4.66 ± 2.72^{a} (3.26-11.31)	4.34 ± 0.75^{a} (3.17-5.43)	3.76 ± 0.50^{a} (3.23-4.69)
Ser	5.37 ± 0.36^{c} (4.92-5.78)	4.75 ± 0.50^{b} (4.03-5.41)	3.60 ± 0.63^{a} (2.88-4.50)	4.82 ± 0.50^{b} (4.33-5.79)
Pro	5.68 ± 2.33^{b} (0.57-8.25)	0.67 ± 0.31^{a} (0.40-1.24)	6.62 ± 2.35^{b} (3.57-10.46)	6.53 ± 3.92^{b} (1.96-11.75)
Met	3.03 ± 0.32^{a} (2.55-3.39)	3.741 ± 0.32^{b} (3.26-4.22)	4.15 ± 0.87^{b} (3.33-5.52)	2.99 ± 0.47^{a} (2.43-3.82)
Ile	4.15 ± 0.41^{a} (3.31-4.58)	4.39 ± 0.49^{a} (3.95-5.28)	4.36 ± 0.37^{a} (3.76-4.89)	4.49 ± 0.31^{a} (3.96-4.87)
Leu	7.25 ± 1.21^{c} (4.55-8.45)	5.22 ± 1.13^{b} (3.80-6.46)	4.26 ± 0.52^{a} (3.58-4.91)	8.61 ± 0.70^{d} (7.77-9.51)
Phe	7.07 ± 0.49^{a} (6.33-7.80)	7.69 ± 1.18^{a} (5.88-9.09)	7.24 ± 1.49^{a} (5.36-9.19)	6.46 ± 1.21^{a} (5.22-8.53)
Lys	5.32 ± 2.54^{a} (3.65-9.75)	4.78 ± 1.60^{a} (3.10-7.39)	3.86 ± 1.56^{a} (2.27-7.00)	3.93 ± 0.56^{a} (2.92-4.55)
His[1]	3.16 ± 0.35^{b} (2.42-3.53)	3.91 ± 0.63^{b} (3.05-4.95)	3.17 ± 1.35^{b} (0.00-4.37)	2.27 ± 0.54^{a} (1.71-3.42)
Arg[2]	5.89 ± 3.70^{c} (0.00-8.78)	2.39 ± 2.03^{b} (0.00-4.65)	0.00 ± 0.00^{a} (0.00-0.00)	3.05 ± 0.76^{b} (2.12-4.61)
Tyr	7.76 ± 0.91^{b} (6.99-9.72)	10.04 ± 1.31^{c} (8.53-11.84)	10.29 ± 1.290^{c} (8.12-12.74)	6.08 ± 0.852^{a} (4.97-7.67)
Val	7.76 ± 1.323^{ab} (4.79-8.60)	8.75 ± 0.739^{b} (7.20-9.46)	7.33 ± 0.70^{a} (6.56-8.41)	7.26 ± 2.03^{a} (2.65-9.01)
Cys	4.55 ± 0.70^{ab} (3.52-5.82)	8.50 ± 1.69^{b} (7.08-11.81)	9.22 ± 1.45^{a} (7.70-11.36)	4.19 ± 0.90^{a} (3.10-6.12)
GABA	0.92 ± 0.48^{a} (0.06-1.35)	3.37 ± 2.19^{b} (0.04-6.02)	2.47 ± 2.03^{ab} (0.12-5.85)	6.77 ± 1.26^{c} (5.48-8.85)

[1]His was not detected in one FMV. [2]Arg was not detected in two SAVs, three ZAVs and all FMVs. Different superscript letters indicate significant difference in the same row, $p < 0.05$.

Table S7 The relative percentage of organic acid contents in SAV, ZAV, FMV and SBV.

Organic acids	Relative percentage (%) (Range (%))			
	SAV (n=8)	ZAV (n=8)	FMV (n=8)	SBV (n=8)
Oxalate acid	0.45 ± 0.34^{ab} (0.08-0.90)	1.14 ± 0.46^{c} (0.46-1.74)	0.76 ± 0.50^{bc} (0.07-1.67)	0.24 ± 0.14^{a} (0.04-0.46)
Tartaric acid	1.01 ± 0.94^{b} (0.02-2.08)	0.57 ± 0.50^{ab} (0.11-1.58)	0.08 ± 0.10^{ab} (0.00*-0.32)	0.53 ± 0.93^{a} (0.01-2.25)
Formic acid	0.28 ± 0.34^{a} (0.07-0.82)	0.77 ± 0.32^{b} (0.28-1.09)	0.21 ± 0.19^{a} (0.06-0.57)	0.38 ± 0.28^{a} (0.06-0.80)
Malic acid[1]	0.02 ± 0.03^{a} (0.00-0.07)	0.02 ± 0.03^{a} (0.00*-0.07)	0.00 ± 0.00^{a} (0.00-0.00)	0.07 ± 0.02^{b} (0.05-0.11)
Pyruvic acid[2]	0.22 ± 0.19^{a} (0.00-0.67)	0.77 ± 1.08^{ab} (0.00-2.97)	0.68 ± 0.44^{ab} (0.08-1.26)	0.94 ± 0.39^{b} (0.13-1.27)
Lactic acid	24.07 ± 5.12^{b} (16.92-33.91)	22.25 ± 6.94^{b} (5.50-26.44)	7.55 ± 2.64^{a} (3.79-12.20)	65.59 ± 12.94^{c} (42.69-78.34)
Acetic acid	69.80 ± 6.58^{b} (56.16-78.10)	69.29 ± 6.77^{b} (63.06-85.46)	89.93 ± 2.89^{c} (84.96-94.41)	28.72 ± 13.88^{a} (12.90-50.82)
Pyroglutamic acid[3]	1.08 ± 1.14^{b} (0.00-2.51)	0.00 ± 0.00^{a} (0.00-0.00)	0.29 ± 0.14^{a} (0.11-0.57)	1.17 ± 0.62^{b} (0.00-2.09)
Citric acid[4]	2.02 ± 2.18^{b} (0.00-4.67)	4.34 ± 2.05^{c} (1.68-7.66)	0.00 ± 0.00^{a} (0.00-0.00)	0.00 ± 0.00^{a} (0.00-0.00)
Succinic acid	1.13 ± 1.61^{ab} (0.02-4.81)	0.85 ± 0.37^{ab} (0.28-1.47)	0.49 ± 0.10^{a}(0.27-0.60)	2.37 ± 2.33^{b} (0.32-0.60)
Fumaric acid[5]	0.002 ± 0.002^{c} (0.00-0.01)	0.006 ± 0.003^{b} (0.00-0.01)	0.009 ± 0.001^{a} (0.007-0.01)	0.002 ± 0.003^{c} (0.00-0.01)

[1]Malic acid was not detectable in all FMVs and four SAVs. [2]Pyruvic acid was not detected in one SAV sample and three ZAVs. [3]Pyroglutamic acid was not detected in four SAVs, one SBV and all ZAVs. [4]Citric acid was not detected in four SAVs, all FMVs and all SBVs. [5]Fumaric acid was not detected in three SAVs and six SBVs. [*]The value was less than 0.01. Different superscript letters indicate significant difference in the same row, $p < 0.05$.

Table S8 The numbers of all the compounds in the four vinegars.

Number	Compounds
NO_1	Oxalic acid
NO_2	Tartaric acid
NO_3	Formic acid
NO_4	Malic acid
NO_5	Pyruvic acid
NO_6	Lactic acid
NO_7	Acetic acid
NO_8	Pyroglutamic acid
NO_9	Citric acid
NO_10	Succinic acid
NO_11	Fumaric acid
NO_12	Asp
NO_13	Thr
NO_14	Ser
NO_15	Glu
NO_16	Gly
NO_17	Ala
NO_18	Cys
NO_19	Val
NO_20	Met
NO_21	Ile
NO_22	Leu
NO_23	Tyr
NO_24	Phe
NO_25	GABA
NO_26	Lys
NO_27	His
NO_28	Arg
NO_29	Pro

NO_30	3-Methylbutyl acetate
NO_31	Ethyl Acetate
NO_32	Hexanoic acid ethyl ester
NO_33	Acetic acid hexyl ester
NO_34	Ethyl L(-)-lactate
NO_35	2-Furanmethanol acetate
NO_36	Ethyl 2-hydroxyisocaproate
NO_37	Diethyl butanedioate
NO_38	Acetic acid benzyl ester
NO_39	2-Methylpropyl acetate
NO_40	Ethyl phenylacetate
NO_41	2-Phenylethyl propionate
NO_42	Acetic acid 2-phenylethyl ester
NO_43	Ethyl hexadecanoate
NO_44	Methylpyrazine
NO_45	2,6-Dimethylpyrazine
NO_46	2,3-Dimethylpyrazine
NO_47	2,3,5-Trimethylpyrazine
NO_48	2,3-Dimethyl-5-ethylpyrazine
NO_49	2,3,5,6-Tetramethylpyrazine
NO_50	2,3,5-Trimethyl-6-ethylpyrazine
NO_51	Eucalyptol
NO_52	Benzyl alcohol
NO_53	2,3-Butanediol
NO_54	Phenylethyl alcohol
NO_55	2-Furanmethanol
NO_56	2-Ketobutan-3-yl acetate
NO_57	Acetic acid
NO_58	Propanoic acid
NO_59	2-Methylpropanoic acid
NO_60	Butanoic acid
NO_61	3-Methylbutanoic acid

NO_62	Pentanoic acid
NO_63	Hexanoic acid
NO_64	Octanoic acid
NO_65	4-Hexyl-2,5-dioxofuran-3-acetic acid
NO_66	Furfural
NO_67	1-(2-furanyl)-Ethanone
NO_68	2,2-Methylenebisfuran
NO_69	2-Acetyl-5-methylfuran
NO_70	2-Methoxyphenol
NO_71	2-methoxy-4-methylphenol
NO_72	2-Methoxy-5-methylphenol
NO_73	2-Methylphenol
NO_74	Phenol
NO_75	4-Ethyl-2-methoxyphenol
NO_76	4-Ethylphenol
NO_77	2-Ethylphenol
NO_78	Nonanal
NO_79	Benzaldehyde
NO_80	5-Methyl-2-furfuralaldoxime
NO_81	Beta-cyclocitral
NO_82	Benzeneacetaldehyde
NO_83	2-Methyl-3(2-furyl)acrolein
NO_84	2-Phenylcrotonaldehyde
NO_85	1H-Pyrrole-2-carboxaldehyde
NO_86	5-Methyl-2-phenyl-2-hexenal
NO_87	Butanal
NO_88	3-Hydroxy-2-butanone
NO_89	1,3,3-Trimethyl-bicyclo-heptan-2-one
NO_90	4-(2-furanyl)-3-Buten-2-one
NO_91	Dihydro-5-pentyl-2(3H)-furanone
NO_92	Styrene
NO_93	1,2,3,4-Tetramethylbenzene

NO_94	1,2,3,5-Tetramethylbenzene
NO_95	4-Ethyl-1,3-benzenediol
NO_96	1,2-Dihydro-1,1,6-trimethylnaphthalene
NO_97	1-(1H-pyrrol-2-yl)-Ethanone
